# Heparan Sulfate Biosynthesis and Sulfation Profiles as Modulators of Cancer Signalling and Progression

**DOI:** 10.3389/fonc.2021.778752

**Published:** 2021-11-11

**Authors:** Catarina Marques, Celso A. Reis, Romain R. Vivès, Ana Magalhães

**Affiliations:** ^1^ Instituto de Investigação e Inovação em Saúde (i3S), Universidade do Porto, Porto, Portugal; ^2^ Instituto de Patologia e Imunologia Molecular da Universidade do Porto (IPATIMUP), Porto, Portugal; ^3^ Programa Doutoral em Biologia Molecular e Celular (MCbiology), Instituto de Ciências Biomédicas Abel Salazar (ICBAS), Universidade do Porto, Porto, Portugal; ^4^ Instituto de Ciências Biomédicas Abel Salazar (ICBAS), Universidade do Porto, Porto, Portugal; ^5^ Faculdade de Medicina da Universidade do Porto (FMUP), Porto, Portugal; ^6^ Univ. Grenoble Alpes, CNRS, CEA, IBS, Grenoble, France

**Keywords:** cancer, cell signalling, glycosyltransferases, glycosaminoglycan, heparan sulfate, heparan sulfate binding epitopes, sulfotransferases

## Abstract

Heparan Sulfate Proteoglycans (HSPGs) are important cell surface and Extracellular Matrix (ECM) maestros involved in the orchestration of multiple cellular events in physiology and pathology. These glycoconjugates bind to various bioactive proteins *via* their Heparan Sulfate (HS) chains, but also through the protein backbone, and function as scaffolds for protein-protein interactions, modulating extracellular ligand gradients, cell signalling networks and cell-cell/cell-ECM interactions. The structural features of HS chains, including length and sulfation patterns, are crucial for the biological roles displayed by HSPGs, as these features determine HS chains binding affinities and selectivity. The large HS structural diversity results from a tightly controlled biosynthetic pathway that is differently regulated in different organs, stages of development and pathologies, including cancer. This review addresses the regulatory mechanisms underlying HS biosynthesis, with a particular focus on the catalytic activity of the enzymes responsible for HS glycan sequences and sulfation motifs, namely D-Glucuronyl C5-Epimerase, N- and O-Sulfotransferases. Moreover, we provide insights on the impact of different HS structural epitopes over HSPG-protein interactions and cell signalling, as well as on the effects of deregulated expression of HS modifying enzymes in the development and progression of cancer. Finally, we discuss the clinical potential of HS biosynthetic enzymes as novel targets for therapy, and highlight the importance of developing new HS-based tools for better patients’ stratification and cancer treatment.

## Introduction

Heparan Sulfate Proteoglycans (HSPGs) are important glycoconjugates ubiquitously expressed on cells glycocalyx and Extracellular Matrix (ECM), as well as in secreted extracellular vesicles (1–3). These macromolecules control several regulatory mechanisms related to the formation of extracellular gradients, cellular growth and proliferation, cell adhesion, migration and invasion, membrane trafficking and angiogenesis (4, 5). By interfering with these cellular events, HSPGs display important functions both in physiology and pathology, controlling embryonic development, ECM assembly and maintenance, tissue remodelling, metabolism homeostasis, pathogen invasion and inflammation (6–12). Particularly in cancer, HSPGs and HS chains hold very relevant roles in the development and progression of the disease. Their interaction with different ligands and structural proteins, and modulation of several signalling networks, prompts their active participation in cell transformation and proliferation, tumour growth and metastasis, amongst other cell oncogenic events (2, 12, 13), highlighting the fundamental need of further studying HS biosynthesis in the context of cancer.

HSPGs are composed by a core protein to which Heparan Sulfate (HS) polysaccharide chains are covalently attached, and can be differentially classified according to (i) their cellular and subcellular localization; (ii) PGs core protein homology; and (iii) function (14). HS is an anionic, long and linear glycosaminoglycan (GAG) chain consisting of repeating disaccharide units of N-acetyl-glucosamine (GlcNAc) and hexuronic acid residues, that can be either glucuronic acid (GlcA) or its C5 epimer, iduronic acid (IdoA) (1). The biosynthesis of HS chains occurs at the endoplasmic reticulum-Golgi apparatus interface and in the Golgi apparatus, and includes: i) assembly of a GAG-protein linker, which initiates the covalent binding of HS to proteoglycan core proteins; ii) the polymerization of the HS chain; and iii) the structural modification of the elongated chain (8). The first two stages of HS biosynthesis involve the sequential transfer of sugar residues to the growing chain and are catalysed by different glycosyltransferases. The polymerized chain then undergoes maturation by several HS modifying enzymes, including N-Deacetylase/N-Sulfotransferases (NDSTs), D-Glucuronyl C5-Epimerase (GLCE) and different O-Sulfotransferases (2OST, 6OSTs, 3OSTs) (15). Finally, further modifications of HS structure take place post-synthetically, through the action of 6-O-endosulfatases Sulf-1 and Sulf-2, and Heparanase (16, 17). Structural modification reactions catalysed by these enzymes modulate glycan chain length, epimerization and sulfation profiles, resulting in the synthesis of HS chains with extremely high structural variability (8). These features are key for regulating HSPG biological roles, since they dictate HS-protein binding affinity and selectivity. HS sulfation degree and patterns are particularly relevant, giving rise to highly negatively charged regions and further promoting non-covalent ionic bonding between HS and positively charged amino acid residues in multiple protein targets, including transmembrane receptors, ECM structural proteins and soluble molecules (18). These features allow HSPGs to participate in signalling cascades and take over a range of cell regulatory events. However, the molecular mechanisms promoting the synthesis of particular HS structures are still largely unknown. Furthermore, only a few specific HS sequences have been identified as essential motifs for HS-protein interactions. A significant limitation in studies that aim to uncover HS-ligand binding specificities is the frequent use of heparin and/or short oligosaccharides, which only partially mimic HS natural complexity. The structural details of HSPG interactomes remains therefore mostly unexplored.

## HS Biosynthesis and Modification

### Biosynthetic Pathway

The biosynthesis of GAGs, and HS in particular, is not a template-driven process, meaning that the extent of modification reactions that the glycan chains may undergo, and the consequent HS final structural motifs, are not directly encoded in the genome. The current general view is that HS biosynthesis is regulated by the availability and expression levels of the enzymes involved in this pathway, and by the binding specificities of these enzymes towards the sequences that are formed in HS growing chains (19). Hence, the structural features resulting from each stage of HS biosynthesis determine subsequent modification reactions, contributing to the “step-by-step” fine tuning of HS overall structure. However, not all of the HS substrates are equally modified, thus giving rise to a great HS structural variability.

The majority of the enzymes that intervene and control HS biosynthesis are located in the Golgi apparatus and are classified as type II transmembrane proteins (15). A HS biosynthesis model has proposed that these enzymes are in tight interaction with each other, forming a supramolecular complex called “GAGosome”, which ensures quick and concerted reactions towards the formation of the GAG chains (20). However, the regulatory mechanisms underlying the activity of this possible enzymatic machinery complex and its impact in HS high structural variability are not yet fully disclosed.

**Figure 1:**
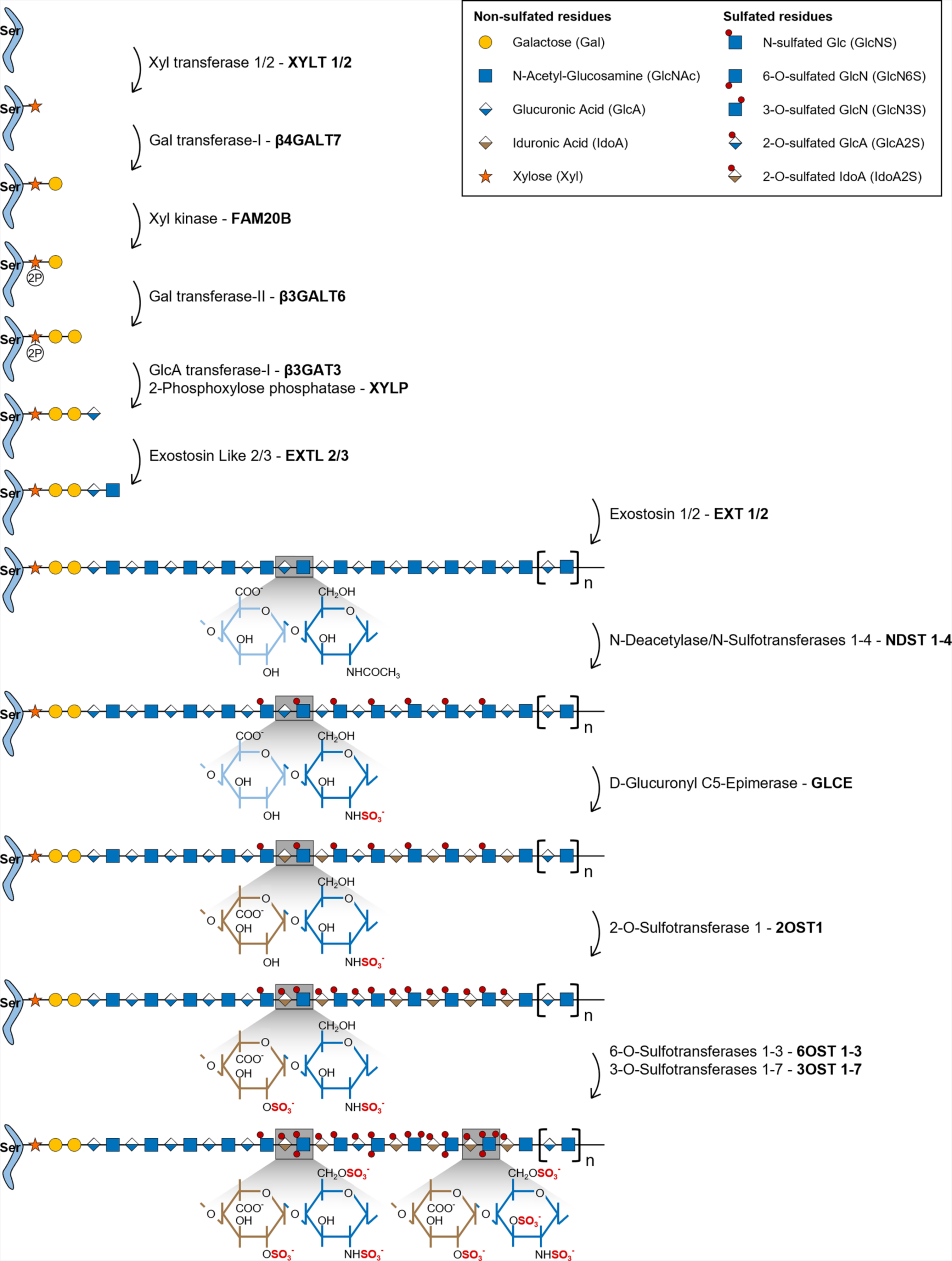
Illustrative representation of the HS biosynthetic pathway and the disaccharide structural changes that occur during each modification reaction. HS biosynthesis comprises the stepwise transfer of different sugar residues that leads to the elongation of HS chains. In the last stage of this biosynthetic pathway, polymerized HS chains undergo multiple modification reactions: i) NDSTs 1-4 catalyse the removal of the acetyl group from GlcNAc residues and the transfer of a sulfate group to free amino groups to form GlcNS; ii) GLCE epimerizes GlcA residues into IdoA; iii) these residues are then targeted by 2OST1, that transfers sulfate groups to the C2-position of IdoA (more rarely GlcA),thereby preventing further epimerization; iv) lastly, 6OSTs 1-3 and 3OSTs 1-7 add sulfate groups to the C6- and C3-positions of GlcN residues, respectively. Chains non-reducing termini are to the right of the saccharide’s sequences. Glycan structures are represented according to the Symbol Nomenclature for Glycans (SNFG) (21).

HS biosynthesis is initiated by the formation of a universal tetrasaccharide linker covalently attached to the core protein of all GAG bearing proteoglycans, including HSPGs. Assembly of this tetrasaccharide linker starts with the transfer of a xylose (Xyl) residue to specific protein serine amino acids by two O-Xylosyltransferases (XYLT1 and XYLT2) (**Figure 1**) (22). This is followed by the sequential transfer of two galactose (Gal) residues, successively added by the Galactosyltransferase-I/β4-Galactosyltransferase 7 (β4Gal-T7) and the Galactosyltransferase-II/β3-Galactosyltransferase 6 (β3Gal-T6), and lastly by the addition of one GlcA residue by the Glucuronyltransferase-I (GlcAT-I), to form the linker GlcAβ1-3Gal-β1-3Gal-β1-4Xyl-β1-O-Ser. During this assembly, transient phosphorylation of the Xyl residue by the kinase FAM20B and dephosphorylation by the 2-Phosphoxylose phosphatase (XYLP) also take place, which enhance the activity of β3Gal-T6, further promoting the maturation of the linker (23, 24). The polymerization of HS chains is then initiated by the transfer of a GlcNAc residue to the tetrasaccharide linker, which is regulated by Exostosin Like 2 (EXTL2) (25) and EXTL3 glycosyltransferases (26), and is followed by further chain elongation promoted by a hetero-oligomeric complex formed by EXT1 and EXT2, which catalyses the alternate transfer of GlcNAc and GlcA residues (**Figure 1**) (27). Polymerized HS chains then undergo extensive processing and modification reactions that give rise to fully mature HS chains, which are the main focus of this review.

### Catalytic Activity of HS Modifying Enzymes

#### *N*-Deacetylation and *N*-Sulfation

During the HS modification stage, native HS polysaccharides, consisting of unmodified repeating GlcNAc residues linked to GlcA, are initially targeted by N-Deacetylase/N-Sulfotransferases 1-4 (NDSTs 1-4), that remove *N*-acetyl groups from GlcNAc residues and transfer a sulfate group to the generated free amino groups to form *N*-sulfated glucosamine residues (GlcNS) (**Figure 1**). This first modification is essential to all the remaining reactions that take place in the Golgi, since most of the modifying enzymes act on GlcNS containing motifs (20). Of note, a small subset of *N*-deacetylated residues do not undergo *N*-sulfation, giving rise to unsubstituted glucosamine molecules (GlcNH_3_), which elicit specific biological functions (1, 19). In vertebrates, NDST1 and NDST2 are the most widely expressed isoforms, occurring in several tissues, while NDST3 and NDST4 are more expressed at the embryonic stage and adult brain (28). The different isoforms also vary in their activity, NDST1 and NDST2 have similar *N*-deacetylase/*N*-sulfotransferase ratio activity, while NDST3 and NDST4 exhibit opposite trends, with NDST3 presenting the lowest sulfotransferase capacity (28) and NDST4 the lowest *N*-deacetylase activity (29) of all four enzymes.

#### Epimerization and 2-*O*-Sulfation

HS chains are subsequently modified by D-Glucuronyl C5-Epimerase (GLCE), which catalyses the epimerization of D-GlcA residues into L-IdoA (**Figure 1**) (30). This enzyme targets specifically GlcA residues located at the reducing side of GlcNS residues and catalyses both irreversible and reversible epimerization, since it can also convert IdoA units back to GlcA (31). This “two-way” catalytic activity was observed through *in vitro* enzymatic assays and it was shown to depend upon HS *N*-sulfation patterns (GlcNS vs GlcNAc content) (32).

2OST1 catalyses then the transfer of sulfate groups to the C2-position of both GlcA and IdoA residues, although with significantly increased efficiency towards the later, displaying around five-fold higher affinity to epimerized IdoA units (**Figure 1**) (33). 2-*O*-sulfation of IdoA residues prevents their reverse epimerization into GlcA, since GLCE cannot use IdoA2S as substrate, which promotes accumulation of these residues on HS chains (34). In addition, it has been proposed that 2-*O*-sulfated GlcA residues (GlcA2S), generated at a smaller extent in HS chains, might result from early 2-*O*-sulfation, prior to the epimerization of GlcA, blocking the subsequent activity of GLCE and conversion of those residues into IdoA (35). Moreover, as a prerequisite for 2OST1 efficient activity, the targeted GlcA or IdoA residues must also be flanked by two GlcNS residues, instead of GlcNAc (36).

These structural alterations that ultimately dictate HS GlcA/IdoA ratio are of extreme relevance for HS functional diversity, since GlcA to IdoA epimerization confers higher structural flexibility to HS chains, which is reflected on HS-protein binding properties (30).

#### 6-*O*-Sulfation and 3-*O*-Sulfation

In the later stages of HS biosynthesis, 6-O-Sulfotransferases 1-3 (6OSTs 1-3) and 3-O-Sulfotransferases 1-7 (3OSTs 1-7) add sulfate groups to the C6- and C3-positions of glucosamine (GlcN) residues, respectively, contributing to the formation of more heterogeneous HS structures (**Figure 1**) (37). 6OSTs have broader substrate recognition and 6-*O*-sulfation activity. Enzymatic assays performed with recombinant mouse 6OSTs 1-3 have shown that all isoforms can transfer sulfate groups to both GlcNAc and GlcNS residues attached to either GlcA or IdoA residues, although they target preferably IdoA-containing disaccharides (38). Furthermore, it was demonstrated that 2-*O*-sulfation of IdoA residues did not affect 6OST activity (38). Interestingly, a different study using heparin-based oligosaccharide libraries to assess 6OST preferred substrates, revealed that both 6OST2 and 6OST3 had higher specificity for oligosaccharides with higher content in 2-*O*-sulfation (39). Habuchi H. et al. have also reported that different 6OST isoforms expressed in mice had different specificities towards varied HS polysaccharide samples depending on their hexuronic acid content: 6OST1 targeted preferentially substrates containing IdoA-GlcNS disaccharide units, 6OST2 could act on both IdoA-GlcNS or GlcA-GlcNS motifs depending on substrate concentration, and 6OST3 utilized both substrates independently of substrate abundance (40). 6OST differential expression over different mouse organs was also reported, which supports tissue-dependent expression and consequent varying effects on HS structural motifs (40). 6-*O*-sulfation contributes to fine-tune the sequential biosynthesis of HS chains, since GlcA epimerization by GLCE is precluded by 6-*O*-sulfation of adjacent GlcN residues (34), and 2-*O*-sulfation of IdoA by 2OST1 is inhibited if the adjacent GlcN residue is 6-*O*-sulfated (36, 41). These data are in line with the described stepwise mechanism underlying HS biosynthesis, further supporting that epimerization and 2-*O*-sulfation reactions usually take place prior to 6-*O*-sulfation (**Figure 1**). More recently, it was uncovered that 6-*O*-sulfated HS oligosaccharides were able to bind to 2OST1, with higher affinity than 2OST1 direct substrates, and inhibit its catalytic activity (42). In light of these results, it was hypothesized that enzymes involved in the HS biosynthetic pathway might be temporarily and/or spatially separated from the intermediate substrates produced downstream on the Golgi. This would explain why HS modified structures do not impair the activity of initially acting sulfotransferases towards new native glycans (42).

Although 3OSTs constitute the largest family of HS sulfotransferases, composed by 7 isoforms (3OST-1, -2, -3a, -3b, -4, -5 and -6), GlcN 3-*O*-sulfation is the least frequent modification (8). The different isoforms exhibit specific binding affinity to GlcN residues linked to different hexuronic acid residues contributing to the functional diversity of HS chains. 3OST1 transfers sulfate groups to GlcNS and GlcNS6S bound to unsulfated GlcA and IdoA units (43, 44). *In vitro* enzymatic assays have revealed that murine 3OST1 activity is specifically inhibited by IdoA2S residues linked to the targeted GlcN residues, suggesting an additional layer of regulation, according to which HS 2-*O*-sulfation content impacts 3-*O*-sulfation patterns and overall levels (44). 3OST2 transfers sulfate groups to GlcNS residues attached to either GlcA2S or IdoA2S, while 3OST3A and 3OST3B isoforms act on GlcNS linked to IdoA2S (45). Later, it was also shown that the 3OST3A isoform can utilize substrates on rarer regions of HS motifs, including *N*-unsubstituted glucosamine (GlcNH_3_) and *N*-unsubstituted 6-*O*-sulfated glucosamine (GlcNH_2_6S) residues linked to IdoA2S at the non-reducing end (46). 3OST4 and 3OST6 catalyse 3-*O*-sulfation on similar disaccharides targeted by 3OST3 isozymes (GlcN residues linked to IdoA2S residues at the non-reducing site) (47, 48). Lastly, 3OST5 was revealed to have a broader substrate specificity when compared with the other isoforms, targeting both GlcNH_3_ and GlcNS residues at the reducing end of GlcA, IdoA and IdoA2S residues (49).

#### Post-Synthetic Modifications

Once HS chains are fully synthesized, and mature HSPGs are expressed at the cell surface, additional post-synthesis reactions can take place and further modify HS chains, namely 6-*O*-desulfation catalysed by 6-O-endosulfatases Sulf-1 and Sulf-2, and cleavage of the chains by Heparanase, thus generating additional structural diversity with biological relevance. These HS chain edition steps have been described in detail in (8, 17). Interestingly, there is evidence of an interplay between biosynthetic enzymatic activity and post-synthesis modification mechanisms. Lamanna W. C. et al. showed that Sulf-1 and/or Sulf-2 KO mice displayed HS structural changes that were not dependent directly on the Sulfs enzymatic activity, such as altered *N*- and 2-*O*-sulfation, which may be explained by the different HS sulfotransferase expression profile observed in these *in vivo* models (50).

## HS Sulfation Epitopes and Ligand Binding Specificities

**Figure 2:**
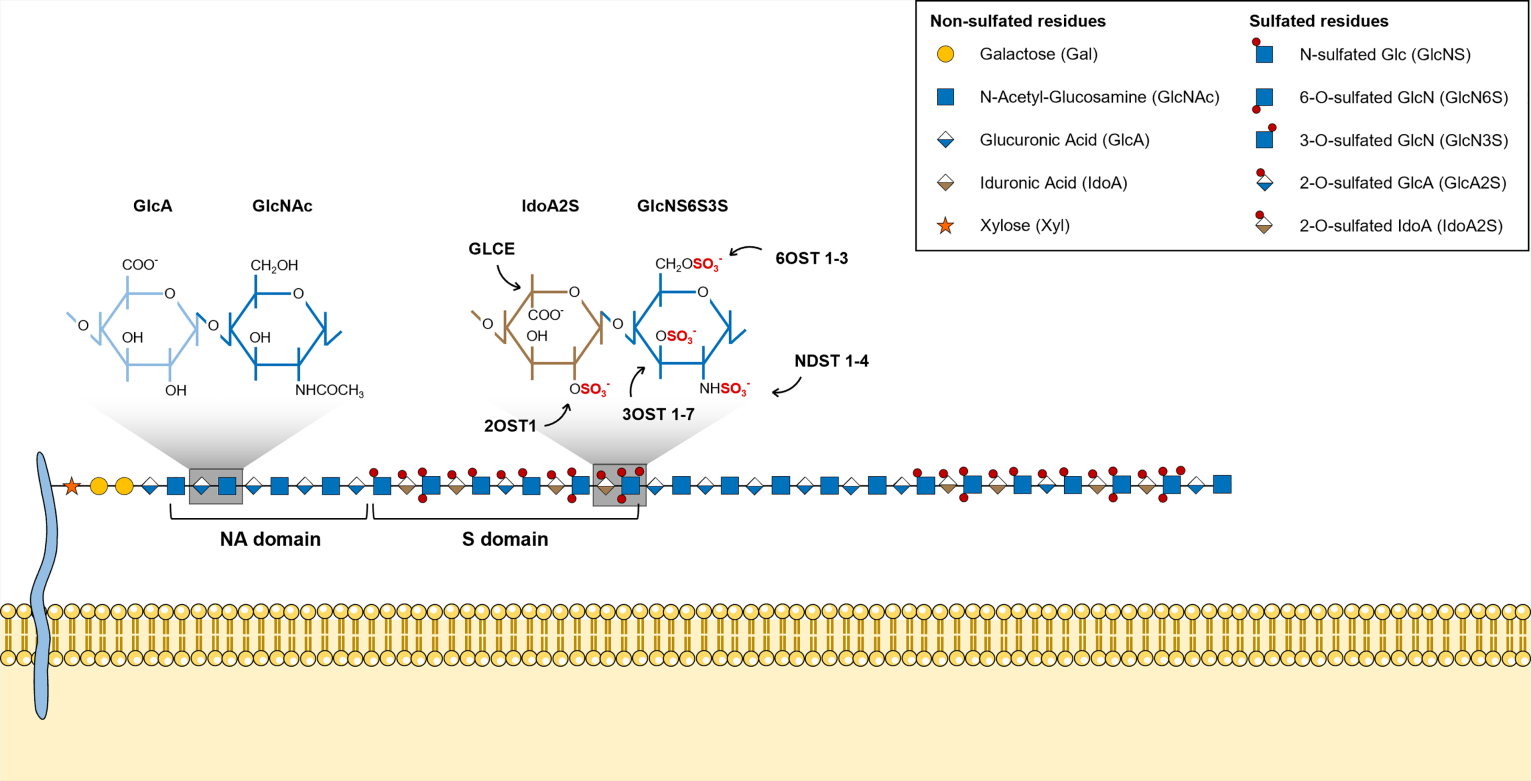
Illustrative representation of a mature HS chain and its structural organization. In mature HS chains, HS disaccharides are organized in two main structural domains: sulfated (S)-domains, enriched in highly modified disaccharides, and *N*-acetylated (NA)-domains, that are mostly composed by non-modified GlcNAc units linked predominantly to GlcA residues. Glycan structures are represented according to the Symbol Nomenclature for Glycans (SNFG) (21).

HS chains feature two main types of structural domains: sulfated (S)-domains, enriched in highly modified disaccharides, i.e. sulfated hexuronic acid units linked to *O*-sulfated GlcNS residues, that are successively intercalated by *N*-acetylated (NA)-domains bearing mostly non-modified GlcNAc units linked predominantly to GlcA residues (**Figure 2**) (8, 51). HS S-domains are responsible for most of the polysaccharide biological activities, their epimerization level and sulfation patterns providing functional diversity. These features indeed modulate HS binding specificities to protein targets, and vary over different organs (52), developmental stages (53–55) and pathologies (56–58). Although most biomolecules interact with the S domains of HS chains, HSPGs can also bind ligands and transduce signal through their protein core (59, 60).

HS chains bind to multiple biologically active molecules, including growth factors, chemokines, cytokines, morphogens, extracellular structural proteins, enzymes involved in different biochemical pathways and transmembrane signalling receptors (5, 61). This prompts cell surface HSPGs to modulate main cellular events, by acting as co-receptors or scaffolds for protein-protein interactions, triggering receptors activation and subsequent signalling transduction, and as important mechanosignalling transducers of extracellular stimuli (13, 62). HSPGs not only enhance the activity of neighbouring receptors expressed on the same cell, but also participate in the transactivation of receptors, including key Receptor Tyrosine Kinases (RTKs), in adjacent cells, mediating cell-cell crosstalk (63). Recently, HSPGs have been shown to play a regulatory role in the activation of calcium channels, and it has been proposed a mechanism dependent on the cytosolic calcium levels, through which HSPGs modulate cells’ cytoskeleton organization and adhesion (64). In the ECM, HSPGs can also function as storage units and contribute to the formation of extracellular gradients of varied soluble molecules, thereby modulating their availability (6).

The impact of HS overall sulfation degree *versus* specific sulfation patterns as determinants for HS-protein binding has been debated over a long time. It has been established that in the majority of cases tissue-dependent HS general sulfation, and sulfation distribution by blocks (S-domains and NA-domains), rather than unique sulfation sequences encoded in HS glycan chains, determine binding affinities (65). The current consensus is that *N*- and 2-*O*-sulfated units are involved in low specificity binding, 6-*O*-sulfated units in intermediate specificity binding and 3-*O*-sulfated and *N*-unsubstituted units are responsible for high specificity binding, which justifies the strong binding overlap of protein ligands towards HS with variable sulfation patterns reported in different HS-binding protein assays.

### Low and Intermediate HS-Ligand Binding Specificities

Although unique and distinctive biologically active HS structural motifs have not been disclosed for most protein-HS interactions, distinct roles of the most common types of sulfation (*N*-, 2-*O*- and 6-*O*-sulfation) have been reported for protein binding affinities and biological activities. Basic Fibroblast Growth Factor (FGF2 or bFGF) was the first growth factor reported to depend upon cell surface HS as a co-receptor required for the formation of a biologically active FGFR-FGF2-HS ternary complex (66, 67). FGFs constitute a large family of mitogens involved in the regulation of cellular migration, proliferation, differentiation, and survival (68). FGF2-HS interaction, in particular, is important not only for receptor dimerization and activation, but also for evasion of degradation by FGF2 molecules (69). Maccarana M. et al. showed that IdoA2S and GlcNS were important units for FGF2 binding, whereas the presence of GlcN6S residues was irrelevant for HS-FGF2 interaction (70). It was later reported that 6-*O*-sulfated residues were required to bridge FGF2 and FGFR, promoting the formation of the full ternary complexes and receptor activation (71–73).

Interestingly, a specific trisaccharide that included both IdoA2S and GlcNS6S residues (IdoA2S–GlcNS6S–IdoA2S) was invariably detected in FGF1 binding sites in HS chains (74). It was demonstrated that the presence of this specific motif repeated in HS polysaccharides contributed to higher FGF1 binding affinity, when compared with HS polysaccharides with increased overall sulfation, and that 6-*O*-sulfation was important for FGF1-HS interaction (74). Overall, HS structural requisites for binding of FGF1 and FGF2 illustrate the consensus on the general role of *N*- and 2-*O*-sulfation in ligand binding and the specificity provided by 6-*O*-sulfation.

Another example illustrating the biological impact of 6-*O*-sulfation in HS binding specificities relates to Vascular Endothelial derived-Growth Factors (VEGF) driven-signalling. VEGFs are key molecules in vasculogenesis and angiogenesis, regulating these events during homeostasis and pathology progression (75). In a recent report, it was revealed that HS chains present on the transmembrane HSPG syndecan-2 formed a ternary complex with VEGFA_165_ and its target receptor VEGFR2. VEGFA_165_ is an important growth factor involved in retinal angiogenesis, and the formation of this complex was shown to enhance significantly VEGFR2 signalling in endothelial cells, when compared to the binary complex VEGFA-VEGFR2 (76). GAG disaccharide analyses revealed high 6-*O*-sulfation in HS of syndecan-2 (76), a structural feature that had been previously shown to be specifically related with the increased VEGFA_165_ binding affinity to HS chains (77).

HSPGs also modulate the activity of morphogens, which are key elements in the cellular events that occur during early development, by providing a scaffold for stabilizing extracellular gradients of these signalling molecules, or acting directly as cell surface co-receptors for receptors activation. Noteworthy, signalling pathways of major HS-binding morphogens including Hedgehog, Bone Morphogenic Protein (BMP) and Wnt, were shown to be impaired by aberrant HS biosynthesis, resulting from the loss of *EXT* gene expression and function (78, 79). HS bioactive domains for Wnt and for a member of the BMP family, BMP-2, have also been previously disclosed. Wnt interaction with HS and subsequent activation were associated mainly with the polysaccharide 6-*O*-sulfation content, and its structural remodelling by the Sulfs (80, 81). The most critical components of BMP-2 HS-binding domain were revealed to be GlcNS residues, while 6-*O*- and 2-*O*-sulfation were also important, but to a lesser extent (82).

### High HS-Ligand Binding Specificities

The most selective interactions take place in regions of HS chains that contain rare modifications, such as GlcNH_3_ and 3-*O*-sulfated GlcN residues (83). The first identified and most studied example of a HS specific saccharide sequence required for protein high specificity binding and activity relates to antithrombin, an important inhibitor of blood coagulation, whose inhibitory activity is potentiated upon binding to heparin (84). Since the late 70s, many studies have been dedicated to disclose a specific binding epitope within heparin responsible for antithrombin interactions and activity, and led to the identification of an antithrombin binding pentasaccharide GlcNAc6S-GlcA-GlcNS3S6S-IdoA2S-GlcNS6S (85, 86). Within this sequence, the GlcNS3S residue is highlighted as a fundamental distinctive structural feature as it is an otherwise rare unit within heparin/HS chains (85, 87).

The binding of Herpes simplex virus type 1 glycoprotein D to cell surface HS, that functions as a critical receptor for the virus entry in host cells, was shown to also depend on specific sites within HS chains that include 3-*O*-sulfation (either IdoA2S-GlcNH_2_3S6S or IdoA2S-GlcNH_2_3S) (88). In addition to its role in antithrombin activity and cellular viral entry, this less frequent type of HS modification has been associated with a few highly selective interactions with other types of bioactive molecules with impact in cell physiology, including the growth factor FGF7 (89), cyclophilin B (90) and the cell surface receptor Neuropilin-1 (91). Lastly, a recent report showed that 3-*O*-sulfation significantly enhances tau binding to cell surface HS, and subsequent tau cellular uptake, ultimately contributing to the prion-like spread of tau pathology in Alzheimer’s Disease (92).

## HS Sulfation PROFILES in Cancer

HSPGs are key players in cancer development and progression, acting as important maestros of cancer cell interaction with the ECM and cancer cell communication, ultimately controlling the tumour microenvironment biochemical and biophysical features (12, 93, 94). The expression of these macromolecules has been shown to be altered in several types of cancer, contributing to the deregulation of different cell events, including proliferation, angiogenesis, adhesion, migration and invasion. The altered cellular levels of HS, resulting from aberrant HSPGs expression and abnormal expression of enzymes involved in HS biosynthesis and editing, are main features heavily involved in cells’ malignant transformation (**Table 1**) (12, 13). Comparative studies have demonstrated aberrant expression of several genes encoding HS biosynthesis machinery in cancer. Transcriptomic and immunohistochemical analyses have been performed in breast (58) and colorectal (113, 114) tumour samples and indicated significant variations in the expression and tissue-distribution of several enzymes involved mostly in HS epimerization and sulfation. In fact, most variations in the expression of HS-related genes detected in several cancer pathologies concern enzymes specifically involved in HS modification reactions that change HS sulfation degree and patterns. This is likely justified by the dependence of the numerous biological roles displayed by HSPGs on these structural features, as discussed previously.

**Table 1:** Aberrant expression of HS modifying enzymes on cancer and its effects on cellular features and patient’s prognosis.

Gene/protein deregulation	Cancer	Altered HS sulfation	Effect on tumour cell and patient’s prognosis	Refs
**↓** *NDST4*	Colorectal cancer (tissue, in higher pathological stages [T3 and T4], and several colorectal cancer cell lines)	◼	Patient’s poor overall survival.	(95)
Ø *Ndst1*	Lung cancer (LLC cells were injected in *Ndst1^f/f^TekCre^+^* mice)	Reduced N-sulfation. Reduced 6-*O*- and 2-*O*-sulfation (mice endothelial cells).	Impaired angiogenesis-related signalling pathways: decreased FGF2- and VEGF-dependent Erk1/2 phosphorylation. Decreased tumour vascularization. Reduced tumour growth.	(96)
**↓** *GLCE*	Breast cancer (tissue and MCF-7 cells)	◼	--	(97, 98)
	Lung cancer (several lung cancer cell lines cells)	◼	--	(99)
⊕ *GLCE*	Breast (MCF7 cells) and small-cell lung cancer (U2020 cells)	◼	Decreased cell proliferation. Supressed the growth of U2020 xenograft tumours.	(98, 99)
**↑** *2OST1*	Prostate cancer (tissue and LNCaP, C4, C4-2, C4-2B cells)	◼	Correlated with metastatic potential.	(100)
Ø *2OST1*	Prostate cancer (LNCaP, C4, C4-2, C4-2B cells)	◼	Decreased cell proliferation and invasion.	(100)
**↓** *2OST1*	Leukaemia (SKM-1 cells under granulocytic differentiation)	◼	Cell growth inhibition and less aggressive phenotypes.	(101)
⊕ *2OST1*	Breast cancer (MDA-MB-231 and MCF-7 cells)	◼	Acquisition of cancer stem cell-like properties.	(102)
⊕ *2OST1*	Breast cancer (MCF-7 and MDA-MB-231 cells)	Increased 2-*O*-sulfation.	Decreased EGFR expression and activation. Upregulated E-cadherin. Promoted cell-cell and cell-ECM adhesion. Inhibited cell migration and invasion.	(103)
**↑** 6OST1	Chondrosarcoma (tissue; correlated with increasing tumour histological grade)	◼	--	(104)
**↑** 6OST2	Chondrosarcoma (tissue; correlated with increasing tumour histological grade)	◼	--	(104)
	Colorectal cancer (tissue and several colorectal cancer cell lines)	◼	--	(105)
**↑** 6OST3	Chondrosarcoma (tissue)	◼	--	(104)
	Breast cancer (T47D, MCF7 and MDA-MB-231 cells)	◼	--	(106)
Ø *6OST3*	Breast cancer (T47D, MCF7 cells)	◼	Increased cell apoptosis and adhesion. Reduced cell proliferation. Reduced cell migration and invasion (except for T47D cells).	(106)
**↓** *3OST2*	Breast, colon, lung and pancreatic cancer (tissue and several breast cancer cell lines)	◼	--	(107)
	Non-small cell lung cancer (tissue and several NSCLC cell lines)	◼	Poor patient’s survival.	(108)
⊕ *3OST2*	Non-small cell lung cancer (H460 and H23 cells)	◼	Reduced cell proliferation, migration and invasion.	(108)
	Breast cancer (MDA-MB-231 cells)	Increased 3-*O*-sulfation.	Increased activation of MAPK and Wnt/β-catenin signalling pathways. Increased cell invasiveness, motility and chemoresistance.	(109)
	Breast cancer (MDA-MB-231 and MCF-7 cells)	◼	Acquisition of cancer stem cell-like properties.	(102)
**↓** *3OST3A*	Breast cancer (luminal-A and triple-negative breast cancer tissues and MCF-7 and MDA-MB-231 cells)	◼	Promoted oncogenic features.	(110)
**↑** *3OST3A*	Breast cancer HER2+ tumours	◼	Decreased disease-free survival.	(110)
⊕ *3OST3B1*	Acute myeloid leukaemia (U937 cells)	◼	Induced cell proliferation. Induced expression and shedding of proangiogenic factors. Promoted pro-angiogenic signalling pathways.	(111)
**↑** *3OST3B1*	Non-small cell lung cancer (tissue and several NSCLC cell lines)	◼	Regulated epithelial-to-mesenchymal transition (EMT).	(112)

↓ downregulation or loss of expression in cancer.

↑ upregulation in cancer.

Ø induced silencing or KO in cancer cell models.

⊕ induced overexpression in cancer cell models.

◼ HS sulfation features were not described.

-- effects on cell behaviour or patient’s prognosis were not reported.

### Sulfotransferase Deregulation and Aberrant HS Sulfation Profiles

Regarding NDST isozymes that act in the initial step of HS modification, namely *N*-deacetylation and *N*-sulfation of GlcN, loss of *NDST4* gene expression in colorectal cancer has been associated with higher pathological stages and patient’s poor prognosis (95). Additionally, Fuster M. M. et al. studied the impact of another NDST isoform, NDST1, on tumour angiogenesis, and showed that endothelial cells isolated from *Ndst1* KO mice synthesized structurally modified HS chains that impaired angiogenesis-related signalling pathways, leading to decreased vascularization of lung tumours and consequent reduced tumour growth (96). Altered expression of GLCE, the epimerase controlling HS GlcA/IdoA ratio, was also reported in cancer. Studies indicated significant decreased expression of *GLCE* in breast tumours (97), even at premalignant stages of the disease, and in lung cancer cells (99). Furthermore, anti-proliferative effects were reported on both breast (98) and small-cell lung (99) cancer cells after induced re-expression of *GLCE*, highlighting this gene as a potential tumour-suppressor gene.

The sulfotransferases that catalyse the more common HS *O*-sulfation reactions, 2OST1 and 6OSTs, have also been implicated in cancer pathology. Gene expression datasets from the Oncomine database were explored and revealed that *2OST1* expression is upregulated in prostate carcinoma and functional assays showed that it correlates with cell proliferation and invasion capabilities, known to enhance cancer cells metastatic behaviour (100). In the same line, but in a different model, it was demonstrated that downregulation of *2OST1*, and concomitant change in HS structural patterns, accompanied the granulocytic differentiation of SKM-1 leukaemia cells and were associated with cells’ growth inhibition and less aggressive phenotypes (101). In breast cancer, *2OST1* expression was shown to promote the acquisition of cancer stem cell-like properties, by activating stemness-associated signalling pathways (102). Additionally, and contradicting the previously reported *2OST1* “tumour promoter gene”-like features, upregulation of *2OST1* in breast cancer cell lines was shown to promote cell-cell and cell-ECM adhesion and to inhibit cell migration and invasion capabilities (103). These effects were associated with increased HS 2-*O*-sulfation and subsequent altered growth factor binding affinities, decreased expression and activation of the Epidermal Growth Factor Receptor (EGFR), which impaired different signalling pathways that modulate cancer cell invasiveness, and increased expression of E-cadherin (103).

Regarding 6OSTs, Waaijer C. et al. reported upregulated expression of 6OST1 and 6OST2 during chondrosarcoma progression, correlated with increasing tumour histological grade, higher levels of 6OST3 in most of the analysed cartilage tumours, and higher levels of 6-*O*-sulfated HS disaccharides in high-grade chondrosarcoma cell lines (104). Similarly, the isoforms *6OST2* and *6OST3* were also found to be overexpressed in colorectal (105) and breast (106) cancer, respectively. *6OST3* expression in particular was shown to influence breast tumour cell growth, invasion and migration (106). HS 6-*O*-sulfation content was also shown to be augmented in the ovarian endothelium of patients with advanced stage ovarian cancer (115). In agreement with this work, it was later disclosed the impact of HS 6-*O*-sulfation content, majorly determined by 6OST isozymes, on ovarian cancer angiogenesis (116). In this work, Cole C. L. et al. showed that HS 6-*O*-sulfation stimulates the heparin-binding EGF-like growth factor (HB-EGF)-dependent activation of cell surface receptor EGFR, which leads to increased expression of angiogenic cytokines (interleukin 6, interleukin 8 and FGF2) by ovarian tumour cells (116). HS post-biosynthesis editing by 6-O-endosulfatases represents an additional regulatory mechanism frequently altered in different tumour models, further emphasizing the role of polysaccharide altered 6-*O*-sulfation in cancer (16, 117).

Lastly, aberrant expression of 3OSTs, which catalyse the rarest HS modification, was also reported in various human cancers, suggesting an important role in cell malignant transformation and tumour cells’ functional features. Miyamoto K. et al. described hypermethylation of 5’ region of the *3OST2* gene and consequent loss of its expression in human primary breast, colon, lung and pancreatic cancers (107). Later, Kumar A. V. et al. have taken this premise and further investigated the possible functions of *3OST2* in tumorigenesis (109). These authors reported that re-expression of this gene in a highly invasive breast cancer cell line augmented the activation of Mitogen-Activated Protein Kinase (MAPK) and Wnt/β-catenin signalling pathways, which promoted breast cancer cell invasiveness, motility and chemoresistance (109). Additionally, *3OST2* expression in breast cancer cells was shown to be related with acquisition of a cancer stem cell-like phenotype (102). Hypermethylation and silencing of *3OST2* was also reported in non-small cell lung cancer, and it was associated with poor overall patient survival. Hwang J. A. et al. induced exogenous expression of this gene in two lung cancer cell lines and observed reduced cells’ migration and invasion capabilities, as well as their lower proliferation rate, supporting that *3OST2* hypermethylation might promote lung tumorigenesis (108). Regarding different isoforms, *3OST3A* was revealed to be epigenetically repressed in breast cancer cell lines representative of distinct molecular subgroups, and it was shown to induce opposite effects, either oncogenic or tumour-suppressive, depending on tumour cell phenotypes (110). Moreover, *3OST3B1* expression was associated to acute myeloid leukaemia progression, by inducing expression and shedding of proangiogenic factors (111), and, more recently, it was found to be upregulated on non-small cell lung cancer and to regulate epithelial-to-mesenchymal transition (112). Interestingly, the telomerase protein TRF2, whose expression is upregulated in tumour cells and associated to suppression of immune response, was shown to regulate *3OST4* expression and to prevent natural killer (NK) cell recruitment and promote cancer immune escape (118, 119).

Together with changes in HS modifying enzymes, altered HS sulfation levels and structural conformations have also been studied and described in different types of cancer, in an attempt to profile specific sulfation patterns as distinctive traits in cancer pathologies that can potentially serve as predictive biomarkers.

GAG disaccharide profiling of cancer patients’ tissue samples has revealed decreased HS 6-*O*-sulfation and total *O*-undersulfation in Hepatocellular cancer (120) and overall decreased HS sulfation, both *N* and *O*, in renal cell carcinoma, mainly due to lower levels of mono 6-*O*-sulfated disaccharides, and all disulfated disaccharides (121). HS sulfation was also shown to vary in breast cancer but in a cell dependent manner. As such, it was shown that MCF7 breast cancer cells, expressing oestrogen and progesterone receptors, produce HS with reduced content in 2-*O*-sulfated disaccharides and enriched in 6-*O*-sulfation. In contrast, HS isolated from MDA-MB-231 and HCC38 cells, triple-negative for oestrogen and progesterone receptors, as well as human epidermal growth factor receptor-2 (HER2), were more 2-*O*-sulfated and less 6-*O*-sulfated, both compared with a non-tumorigenic human mammary gland epithelial cell line (122).

On the other hand, data regarding 3-*O*-sulfation levels in tumours is very scarce. This is mainly due to the shortage of commercially available 3-*O*-sulfated disaccharide standards that can be used for comparison in HS disaccharide structural analyses, as well as the low susceptibility of 3-*O*-sulfated disaccharides to the currently used Heparinases (I, II and III) for exhaustive HS depolymerisation, which is a prerequisite for this type of analyses.

## Targeting of HS and HS Biosynthetic Enzymes

**Figure 3:**
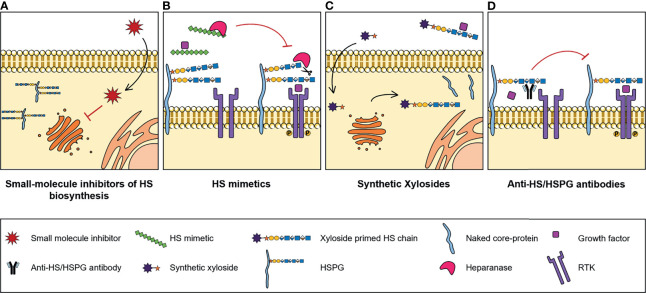
Illustrative representation of the mechanisms of action of four types of molecules developed to target HS biosynthesis and HS-protein interactions, with potential for cancer therapy: **(A)** Small-molecule inhibitors of HS biosynthesis. Cell-permeable, small-molecule compounds with inhibitory activity against GAG biosynthesis enzymes, that impair glycans’ biosynthesis and sulfation. **(B)** HS mimetics. HS-like compounds that compete with HS chains for the binding of enzymes, like Heparanase, or HS-binding proteins, like growth factors, blocking HS-protein interactions and inhibiting the formation of important cell signalling complexes. **(C)** Synthetic xylosides. Synthetic primers for protein-free GAG biosynthesis, that interfere with the GAGosylation of native PGs’ core protein, and when secreted into de extracellular environment, compete with endogenous GAGosylated PGs for the binding of biologically active ligands. **(D)** Anti-HS/HSPG antibodies. Antibody molecules developed to target HS and HSPGs and to inhibit HS-ligand interactions and downstream signalling cascades.

Numerous studies reported abnormal expression of HSPGs and HS biosynthesis enzymes in a myriad of different types of cancers, associated with tumour cell transformation, disease progression and patient outcomes. These may therefore have a significant clinical potential as molecular biomarkers to improve cancer diagnosis and prognosis, and as targets for novel treatment strategies. In addition, and considering the important biological roles displayed by HS chains and how these polysaccharides impact cell malignant features, pharmaceutical strategies that also target HS functions and HS-protein interactions are being developed to tackle cancer pathologies. These strategies include small-molecule inhibitors of HS biosynthesis, HS mimetics, synthetic xylosides and anti-HS/HSPG antibodies (**Figure 3**).

There are also several therapeutical strategies in pre-clinical or clinical studies that target post-biosynthesis editing mechanisms involving the activity of Sulfs and Heparanase, which have been thoroughly reviewed in (16, 17). Still, these are out of the scope of this review, whose primary focus is on the role and activity of HS biosynthesis enzymes, mainly HS sulfotransferases, and on HS-ligand interactions, and therefore have not been further discussed in the following sections.

### Small-Molecule Inhibitors of HS Biosynthesis

Despite the known role of HS sulfotransferases in determining HS conformations and interacting partners, which influences cell signalling networks, the targeting of these enzymes has been considerably less explored for innovative therapeutic strategies. Some reports have focused on the use of small molecules to manipulate the binding and/or activity of HS sulfotransferases over the last years (**Figure 3A**). In one of these projects, Byrne D. P. et al. screened several small-molecules from a Public Kinase Inhibitor Set library, and validated cell permeable compounds as probes with inhibitory activity against 2OST, namely three polyanionic compounds - suramin, aurintricarboxylic acid and rottlerin - and one oxindole RAF kinase inhibitor - GW407323A (123). In addition, a new approach for quicker, reproducible and cheaper detection of substrate sulfation was employed. This sulfation detection strategy is based on microfluidics and differential scanning fluorimetry, and in the future it could be applied for the discovery of new inhibitory compounds against other sulfotransferases, like 6OSTs and 3OSTs (123).

More recently, it was reported a methodology for transient and reversible inhibition of HS biosynthesis also involving a small cell-permeable compound, the tetra-acetylated N-azidoacetylgalactosamine (Ac_4_GalNAz). Maciej-Hulme M. *et al.* have shown that the treatment of Chinese hamster ovary cells with Ac_4_GalNAz induces early termination of HS elongation, reducing HS levels and length, potentially by interfering with the activity of NDSTs (124). In a first instance these types of tools will be extremely relevant to further study HS and HS-sulfation dependent cell mechanisms and impact in cancer pathology, and in the long-term these could potentially be applied in cancer research and therapy.

### HS Mimetics

HS mimetics are synthetic and homogenous molecules, that can either be saccharide or non-saccharide based, with great potential as anti-cancer agents. These molecules function by competing with HS native chains and blocking HS-protein interactions (**Figure 3B**), and a few of them either have been or are currently under clinical trials, namely CX-01 (ODSH), Roneparstat (SST0001), Necuparanib (M402), Muparfostat (PI-88) and Pixatimod (PG545) (125). A great variety of these HS mimetics inhibit either Heparanase and/or Sulfs activity and directly target HS-growth factor interactions, and their specific features and functions have been recently reviewed in (126). Furthermore, it has been reported that these mimetics may also inhibit the activation of RTKs. For example, Roneparstat was shown to inhibit FGF, Insulin Growth Factor (IGF), EGF and Platelet-Derived Growth Factor (PDGF) receptors expressed in sarcoma cells (127).

In addition to these widely studied HS mimetics, several different HS-like compounds have been developed and/or discovered and studied specifically due to their ability to interfere with HS mediated signalling cascades, that are yet to be further explored in clinical studies.

Sutton A. et al. revealed the anti-tumour effects of two synthetic sulfated polymers HS mimetics, OTR4120 and OTR4131, in hepatoma cells. These compounds interfere with a HS-mediated signalling pathway regulated by the CC-chemokine Regulated upon Activation, Normal T Cell Expressed and Presumably Secreted (RANTES), also referred to as CCL5, which was shown to induce cell migration and invasion in a HS-dependent manner (128). The G2.2, another synthetic sulfated non-saccharide HS mimetic, has also been evaluated in regard to its anti-tumour activity and it was shown to selectively target and reduce colorectal cancer stem cells, by inducing the activation of p38 MAPK and impairing cell self-renewal, ultimately reducing tumour growth (129). In a different study, Shanthamurthy C. D. et al. synthesized a highly sulfated IdoA-based oligosaccharide as a novel HS mimetic to target chemokines and modulate its activity in cancer progression, and reported its high binding affinity towards several homeostatic and inflammatory chemokines. Additionally, they showed that this HS mimetic, potentially by binding to CCL2, inhibits breast cancer CCL2-mediated cell proliferation and CCR2/CCL2- mediated cell migration, and it reduces cell invasiveness (130). More recently, Jain P. et al. have resorted to a library of HS tetrasaccharide ligands with varying sulfation patterns and high-throughput array binding assays to further address HS binding specificities, and validated two HS analogues, HT-2,6S-NAc and HT-6S-NAc, as potential ligands to target VEGF_165_-mediated cellular events, as these were shown to not only bind with high affinity to the mentioned growth factor but also to inhibit endothelial cells’ VEGF_165_-induced proliferation, migration and tube formation, which are known to be relevant tumour related features (131).

### Synthetic Xylosides

Another promising tool for cancer therapy pertains to the use of synthetic xylosides. These molecules comprise a xylose residue linked to an aglycone group and serve as primers for protein-free GAG biosynthesis in the Golgi. Synthetic xylosides compete with PGs’ core protein for biosynthetic enzymes, impairing the elongation and modification of GAG chains attached to PGs, while on the other hand the xyloside primed GAG chains secreted into the extracellular environment also compete with endogenous PG-linked GAGs for binding of different ligands (**Figure 3C**) (132).

The use of synthetic xylosides with HS priming activity was shown to effectively inhibit several tumour-related cellular events. Raman K. et al. revealed significant reduction of the invasive capabilities of glioma cells treated with click-xylosides (133). It has also been reported the anti-angiogenic efficacy of two different fluoro-xylosides that by inhibiting HS synthesis in endothelial cells, ultimately inhibit tumour cells’ angiogenesis (134), which is in agreement with the previously mentioned role of HSPGs as important binding partners and co-receptors of pro-angiogenic factors, including VEGF and FGF. More recently, novel xyloside-derived compounds with potential to be used in therapy for glioblastoma were identified due to their ability of impairing endogenous GAG biosynthesis by binding to XYLT1 and β4GAL T7 active sites, and consequently decreasing glioblastoma cell viability (135). In a different study, Mani K. et al. demonstrated that the use of the synthetic xyloside 2(6-hydroxynaphthyl)-β-D-xylopyranoside inhibited tumour cell growth *in vitro*, in human lung and hepatocellular carcinoma, and SV40-transformed mouse embryonic fibroblasts 3T3 cells, as well as *in vivo*, in human bladder carcinoma cells (136). However, unlike the previously mentioned studies, they hypothesised that this anti-proliferative effect, observed at low xyloside doses, was due to the accumulation of antiproliferative products resulting from degradation of the xyloside-primed HS chains inside the cells, rather than competition with PG core protein or PG-linked GAGs for HS biosynthesis or ligand-binding, respectively (136).

HSPGs expressed at cell glycocalyx also function as internalizing receptors for extracellular vesicles (EVs). EVs are cell-derived vesicles that mediate intercellular communication and display crucial roles in a multitude of physiological and pathophysiological processes. In recent years, it has been suggested that EVs released by tumour cells play an important role in the establishment of premetastatic niches and in metastasis. Christianson H. C. et al. have shown that treatment of glioma cells with xylosides inhibited EV cellular uptake and consequently it reduced cell migratory capabilities (137).

Treatment of cancer cells that feature aberrant expression of HS sulfotransferases with xylosides could potentially lessen the synthesis of endogenous PGs modified with HS chains with altered sulfation patterns, diminishing the effects of PG-linked GAG oversulfation on different cell signalling pathways involved in tumorigenesis. Moreover, a higher impact over HS sulfotransferase activities could be achieved by manipulating the structure of the aglycone portion of the synthetic xylosides (138), which could be employed to further increase enzyme affinity towards these synthetic molecules. This type of xyloside dependent HS structural changes were also reported by Chen Y. et al. when comparing the GAG priming efficiency of two synthetic xylosides, 2-naphthyl-β-D-xylopyranoside and its derivative 2-(6-((3-aminopropyl)oxy)-naphthyl)-β-D-xylopyranoside, particularly in terms of sulfation (139).

### Anti-HSPG Antibodies

In addition to the previously mentioned strategies, a different line of investigation has been dedicated to the development and study of molecules that impair HS-ligand interactions by directly targeting HS and HSPGs, namely anti-HSPG antibodies (**Figure 3D**).

Gao W. et al. generated a human monoclonal HS-specific antibody, HS20, targeting HS chains found on glypican-3 (140), a cell surface HSPG known to be highly expressed in hepatocellular carcinoma and associated with patient poor prognosis (141). Treatment of hepatocellular carcinoma cells with HS20 was revealed to block the activation of the HGF/Met pathway, and consequently to inhibit HGF-induced cell migration, motility, and 3D-spheroid formation, as well as *in vivo* liver tumour growth (140).

Syndecan-1 is another cell-surface HSPG that has been revealed to be a potential immunotherapeutic target for cancer therapy, more specifically for multiple myeloma. Jiang H. et al. developed modified NK cells expressing a syndecan-1-specific chimeric antigen receptor (CAR), that showed enhanced *in vitro* and *in vivo* cytotoxicity against syndecan-1 positive multiple myeloma cells, presenting a new possible approach for efficient and specific cancer immunotherapy (142). More recently, it was also developed a new monoclonal anti-syndecan-1 antibody, VIS832, to use in multiple myeloma therapy, and it was shown to induce potent NK cell-mediated antibody-dependent cellular cytotoxicity and macrophage-mediated antibody-dependent cellular phagocytosis against myeloma cells either sensitive or resistant to current therapies (143). In this study it was further demonstrated the efficacy of the VIS832 treatment in a murine model of disseminated human multiple myeloma, both as monotherapy and combined with a proteasome inhibitor (bortezomib) used in therapy (143).

In addition, anti-HSPG antibodies also hold great potential for safer and highly specific drug delivery in cancer immunotherapy. Indatuximab ravtansine (BT062) is an example of such therapeutic approach, as an anti-syndecan-1 monoclonal antibody (nBT062) conjugated with a highly cytotoxic maytansinoid derivative (DM4). This antibody-drug conjugate displayed *in vitro* and *in vivo* anti-tumour activity against multiple myeloma cells expressing syndecan-1, both as monotherapy (144) and in combination with other clinically approved anti-myeloma drugs (145), and more recently it has been under phase I and phase I/IIa clinical trials as monotherapy for relapsed and/or refractory multiple myeloma (146). In a different study, Bosse K. R. et al. have identified glypican-2 as a potential immunotherapeutic target for neuroblastoma due to its significantly higher expression in high-risk neuroblastomas, which was also associated with patient worse overall survival, and developed a highly cytotoxic antibody-drug conjugate that specifically targets glypican-2-expressing neuroblastoma cells (147).

## Conclusion and Perspectives of Future Research

In summary, the roles of HS and HSPGs in physiological events, but most importantly in cancer, highlight these molecules, as well as HS biosynthesis enzymes, as key players during tumorigenic progression. The increasing need for efficient and highly specific biomarkers, and personalized anti-cancer therapies, including new selective drugs, prompts a more in-depth research of these promising tools, inciting further investigation of HS fine structures and their impact in cancer cell behaviour.

Many studies in this field, that have been performed to infer about structural-functional relationships, resorted to artificial *in vitro* models, *via* binding assays and using heavily sulfated heparin molecules and short HS-like oligosaccharides, with the purpose of unravelling bioactive sulfation arrangements and distinctive protein-binding sites within HS chains (70, 148, 149). However, structural differences between these molecules and full-length cellular HS chains might lead to deceitful results, since the use of heavily sulfated heparin might mask specific binding sites and HS oligosaccharides are not presented as embedded in a full-length HS chain. Such assays might thus not accurately represent what occurs in nature (51). Therefore, it has been increasingly important to apply the current knowledge to cell models to assess native GAG biological functions in more complex systems (72, 139), and potentially in disease progression. The great length and high heterogeneity of natural HS chains, whose structures are highly variable, and whose expression is spatially and temporally dependent in organisms, represents a great challenge to this plan (150, 151). Fortunately, the GAG research field is moving at a fast-evolving pace. The development and improvement of robust and sensitive glycoproteomic analytical methodologies combined with computational studies and bioinformatic approaches, such as HS interactome database, will be crucial to assess detailed sequences and conformations adopted by HS glycans implicated in their interactions with proteins in the cell environment (152, 153).

It is also critical to better understand the fine-tuned enzymatic regulatory events that underly HS biosynthesis to elucidate the mechanisms through which endogenous HS modification enzymes regulate tumour cell signalling and disease progression. Overall, the numerous reports describing altered expression of HS sulfotransferases and the abnormal HS sulfation profiles in cancer, further support the clinical potential of these enzymes and structural features as important biomarkers for better diagnosis and prognosis of cancer patients, and as novel targets for improved cancer therapy. However, due to the ubiquitous nature of HSPGs, that exert multiple functions at the physiologic level, it is essential to develop therapeutic approaches targeting specific disease-related modifications, both in terms of HSPG deregulation and GAG conformations. This imposes the challenge of identifying unique cancer Gagosylation signatures to ensure specific targeting and avoid therapy side effects.

## Author Contributions

CM and AM: conceptualization. CM: original draft writing. RV, CR, and AM: manuscript writing and review. All authors contributed to the article and approved the submitted version.

## Funding

This work was funded by FEDER funds through the Operational Programme for Competitiveness Factors-COMPETE (POCI-01-0145-FEDER-007274 and POCI-01-0145-FEDER-028489) and National Funds through the Foundation for Science and Technology (FCT), under the project: PTDC/MED-ONC/28489/2017 (to AM); and COST Action CA18103 INNOGLY: INNOvation with Glycans new frontiers from synthesis to new biological targets (ECOST-STSM-Request-CA18103-45514). CM acknowledges the FCT PhD scholarship 2020.06412.BD. This work was also supported by the “Investissements d’avenir” program Glyco@Alps (ANR-15-IDEX-02) and by grants from the Agence Nationale de la Recherche (ANR-17-CE11-0040 and ANR-19-CE13-0031). IBS acknowledges integration into the Interdisciplinary Research Institute of Grenoble (IRIG, CEA).

## Conflict of Interest

The authors declare that the research was conducted in the absence of any commercial or financial relationships that could be construed as a potential conflict of interest.

## Publisher’s Note

All claims expressed in this article are solely those of the authors and do not necessarily represent those of their affiliated organizations, or those of the publisher, the editors and the reviewers. Any product that may be evaluated in this article, or claim that may be made by its manufacturer, is not guaranteed or endorsed by the publisher.
